# The Formation of Starch-Lipid Inclusion Complex by Enzymatic Hydrolysed Oils

**DOI:** 10.1007/s11130-025-01326-7

**Published:** 2025-03-04

**Authors:** Fatma Nur Akgül, Hümeyra Çetin-Babaoğlu, Sultan Arslan-Tontul

**Affiliations:** Agricultural Faculty, Food Engineering Department, Selçuk University, Konya, 42130 Türkiye

**Keywords:** Resistant starch, RS5, Starch-lipid inclusion complex, Enzymatic hydrolysis

## Abstract

This study aimed to investigate the enzymatic hydrolysis of sunflower, olive, and flaxseed oils and its impact on starch complexation and properties. Enzymatic treatment significantly increased free fatty acid content to 45% without any significant change in fatty acid composition and oxidation precursors. The complexation of high amylose maize starch with hydrolysed flaxseed oil was effective in limiting starch digestion. The highest resistant starch content and expected glycemic index value were determined in starch lipid complex formed by hydrolysed flaxseed oil to be 69.11% and 56.44, respectively. According to XRD patterns, V-type formation was obtained only in complexes formed by hydrolysed oils. Overall, it is concluded that enzymatic hydrolysis of polyunsaturated triglyceride fatty acids to free fatty acids and subsequent complexation with starch can be effective in the production of RS5.

**Supplementary Information:**

The online version contains supplementary material available at 10.1007/s11130-025-01326-7.

## Introduction

Recently, starch-lipid inclusion complex (SLC) has attracted significant research interest as a novel resistant starch (RS). The main reason for this growing interest may be an attempt to find natural ways of making starch less digestible, rather than chemical modifications. In this type of RS, starch fractions of amylose and amylopectin form complexes with saturated or unsaturated fatty acids. Due to the greater ability of amylose to form complexes with lipids compared to amylopectin, these compounds are often referred as amylose-lipid inclusion complexes [1]. As a structural point of view, each of the 6 glucose units of the amylose rotates around a fatty acid, forming a V-type complex. The methylene groups of the fatty acid form the inner surface of the complex, while the hydrophilic -OH groups are located outside the complex. This structure makes the starch resistance to digestive enzymes, increasing the RS content of SLC. This V-type starch formed by complexation is also known as RS5 [2].

According to Web of Science data, studies related to SLC comprise 28% of all studies conducted with RS in the last 5 years [3]. The increasing demand for research on RS5 is due to its superior technological properties compared to other RS types. RS5 has a higher thermal stability, which makes it advantageous for fortifying foods produced by high heat treatments. Additionally, RS5 also requires fewer physical and chemical modification steps than other types of RS. Moreover, the conformational structure of RS5 can revert to its original state after the application of heat [2].

Various fatty acids have been used in the synthesis of SLCs, but the most commonly used are myristic, palmitic, stearic, oleic, linoleic and linolenic acids [4–6]. The fatty acid used in synthesis has a direct effect on the yield of complexes and the amount of RS [5]. While some researchers emphasise that chain length is more influential in the production of complexes [7, 8], others emphasise the degree of unsaturation [5, 9]. Most of the previous studies have been carried out with free forms of fatty acids [4, 10]. Because the ability to form complexes has been reduced by using the triglyceride [11, 12]. However, the use of free fatty acids has certain drawbacks, including the low resistance of unsaturated fatty acids to oxidative reactions, high costs, and limited availability. Therefore, the development of techniques allowing the use of readily available raw oils in RS5 production represents a significant innovation in this field. Such techniques involve subjecting the raw oils intended for RS production to hydrolysis into free fatty acids prior to production. To the best of our knowledge, only one study has been conducted with the specific aim of investigating this phenomenon. In this study, Di Marco, Ixtaina [13] examined the formation of SLC, following the enzymatic hydrolysis of chia seed oil. However, this paper mostly focused on structural characteristics of SLC rather than its digestibility.

The objective of this study is to investigate the potential of sunflower, olive, and flaxseed oils to generate RS5 when combined with high-amylose starch following enzymatic hydrolysis. These raw oils were chosen because they contain major fatty acids of the similar chain length with different levels of unsaturation. Alongside structural and physicochemical analyses, the study also examines starch digestion kinetics and the resulting RS content within the produced complexes.

## Materials and Methods

### Material

In the study, sunflower, olive, and flaxseed oil were acquired from local producers. High amylose corn starch (Hylon VII, containing 72% amylose, < 13% moisture, 0.9% total fat, 0.6% total protein, 86.8% carbohydrate) was supplied by Ingredion Inc. (Illinois, USA). Amano lipase A (EC 3.1.1.3) from *Aspergillus niger* was procured from Sigma (Darmstat, Germany), with an enzymatic activity of ≥ 120,000 U/g. The enzymes used in the starch hydrolysis, namely pepsin (3.4.23.1, > 500 U/mg) were also obtained from Sigma, Darmstat, Germany. Additionally, α-amylase (3.2.1.1., 3000 U/mg), amyloglucosidase (3.2.1.3, 3260 U/ml), and GOPOD (glucose oxidase/peroxidase) were purchased from Megazyme, Ireland. All other chemicals served as analytical standards and were supplied by Sigma, Darmstat, Germany.

### Enzymatic Hydrolysis of Sunflower, Olive and Flaxseed Oil

The oil/water ratio, hydrolysis time, temperature, and pH were optimized through preliminary tests to maximize free fatty acid (FFA) content. All experiments were conducted under identical conditions to ensure reproducibility.

Briefly, 30 mL of oil was suspended in 70 mL of 0.5 M KH₂PO₄ buffer (pH 6.49), and the mixture was homogenized by vigorous stirring. To stabilize the emulsion, 0.1% (w/v) gum Arabic was added and mixed using a magnetic stirrer. The temperature of the mixture was maintained at 40 °C using a magnetic stirrer, and 120 U/mL lipase was added. The mixture was hydrolysed with continuous stirring at 400 rpm for the specified durations: 6 h for flaxseed oil and 8 h for sunflower and olive oils. To minimize variability, the same batch of lipase and reagents was used across all experiments, and the reaction conditions (pH, temperature, and stirring speed) were continuously monitored and adjusted as needed to remain constant. All samples were prepared in duplicate to assess reproducibility.

Following enzymatic hydrolysis, 100 mL of hexane was added to the hydrolysate and mixed thoroughly. The mixture was centrifuged at 3000 rpm for 6 min to separate phases. The upper phase, containing hydrolyzed fatty acids, was collected and evaporated using a rotary evaporator (Rotavapor R300, Buchi, Switzerland) under controlled pressure and temperature. The resulting hydrolysate was stored in amber flasks at refrigerator temperature (4 °C) to minimize oxidation until further analysis.

### Production of Type 5 Resistant Starch (RS5)

To form SLC designated as RS5, a 7.5% (w/v) high amylose corn starch suspension in distilled water was prepared and pregelatinized in a shaking water bath at 95 °C for 30 min. Subsequently, the pregelatinized starch suspension was heated to 121 °C and held for 15 min using an autoclave for complete gelatinisation. After cooling to room temperature, 10% each of raw and hydrolysed oil (v/v) was added to the gelatinised starch and left to mix over a magnetic stirrer for 24 h at ambient temperature. Centrifugation was performed at 3000 rpm for 10 min to collect the SLC. The obtained pellet was washed twice with 50% ethanol and grinded after drying.

### Analysis Performed with Raw and Hydrolysed Oil

Free fatty acid content, peroxide and *p*-anisidin value of raw and non-hydrolysed oil samples were determined according to AOCS [14] with official method number of Ca 5a-40, Cd 8b-90 and Cd 18–90, respectively.

The fatty acid profiles of oils were also fallowed both before and after hydrolysis. A 200 µL oil sample was mixed with 4 mL of HCl: methanol (10% v/v) and incubated at 100 °C for 10 min. Following incubation, 2 mL of hexane was introduced into the sample tubes and vortexed. The methyl esters of fatty acid were collected from the upper layer of tubes and injected to the GC-MS system (7890, Agilent Technologies, CA, USA). A HP-5ms (30 m × 0.25 mm × 0.25 μm, Wilmington, DE, USA) analytic column was used for determination of fatty acid profile and column oven program was applied as fallows: 50 °C for 1 min, heating to 175 °C at 25 °C/min, 175–230 °C at 4 °C/min and held for 15 at 230 °C. Peaks were identified by mass spectral libraries of NIST and Wiley and the result were expressed fallowing the calculation of relative peak areas of chromatogram [13].

### In Vitro Starch Digestion and Expected Glycemic Index (eGI)

The in vitro starch hydrolysis of SCL was determined according to Goñi, Garcia-Alonso [15] and Englyst, Vinoy [16]. 50 mg starch sample was mixed with 10 mL of HCl-KCl solution (pH 1.5). Then 0.2 mL pepsin solution (porcine, 200 U/mL) was added to sample tubes and incubated at 40℃ for 1 h in shaking water bath. After incubation, sample volumes were adjusted to 25 mL of Tris-Maleate buffer (pH 6.9), and 5 mL pancreatic α-amylase solution (2.6 U in the tris-maleate buffer) was added. This solution was incubated at 37℃ for 180 min. During this second incubation period, aliquots of 1 mL were taken every 30 min from 0 to 180 min. To inactivate the enzyme, these aliquots were kept at 100℃ for 5 min and stored at 4℃ until the end of the incubation time. After incubation completed, 3 mL of 0.4 M sodium acetate buffer (pH 4.75) and 60 µL pancreatic amyloglucosidase were added to each aliquot and samples were incubated at 60 °C for 45 min to convert digested starch to glucose. 0.1 mL aliquot was enriched with 3 mL GOPOD (glucose oxidase/peroxidase) and left for final incubation at 45 °C for 20 min. Finally, the glucose concentration was measured at 510 nm, and the measured glucose level was converted into starch by multiplying for 0.9. The rate of starch digestion was expressed as the percentage of total starch hydrolysis at different times of experiment (0, 20, 60, 90, 120 and 180 min. Digestion time and hydrolysed starch was plotted, and a nonlinear starch hydrolysis curve was shaped. To determine the eGI, the C_∞_ and k was calculated from Eq. 1, and they were applied to describe the kinetics of starch hydrolysis.

1 $$\:C={C}_{\infty\:}(1-{e}^{-kt})$$

$$\:C=\:Concentration\:of\:each\:time$$

$$\:{C}_{\infty\:}=\:Concentration\:at\:equilibrium\:$$

$$\:k=\:Kinetic\:constant\:$$

$$\:t=Time$$

The area under the curve (AUC) was calculated from C_∞_ and k constant (Eq. 2).

2 $$\:AUC={C}_{\infty\:}({t}_{f}-{t}_{0}\:)-\frac{{C}_{\infty\:}}{k}\:\left(1-{e}^{-k\left({t}_{f}-{t}_{0}\right)}\right)$$

The hydrolysis index (HI) was calculated as the relation between the AUC of complex and referance. Finally, eGI was calculated by Eq. 3.

3 $$\:eGI=39.71+0.549\:\left(HI\right)$$

Rapidly digested starch content (RDS), slowly digested starch content (SDS) and resistant starch (RS) content of complex were calculated from fallowed equations.

4 $$R D S(\%)=\left(\left(\mathrm{G}_{20}-\mathrm{FG}\right) / \mathrm{TS}\right) \\ \mathrm{x} \\ 0.9 \\\mathrm{x} 100$$

5 $$S D S(\%)=\left(\left(\mathrm{G}_{120}-\mathrm{G}_{20}\right) / \mathrm{TS}\right) \\ t \mathrm{x} \\ 0.9 \\ \mathrm{x} 100$$

6 $$R S(\%)=\left(\left(\mathrm{TS}-\mathrm{G}_{120}\right) / \mathrm{TS}\right) \\ \mathrm{x} \\ 0.9 \\\mathrm{x} 100$$

G_20_ and G_120_ represents glucose concentration at the 20 and 120 min of digestion, while FG means glucose concentration before digestion test. TS means total starch content of sample and accepted as 100.

### Water Binding Capacity (WBC)

The WBC of the complex were determined according to the method of Li, Gao [17]. 0.7 g of SLC was suspended in 10 mL distilled water. The starch suspension was incubated for 1 h at ambient temperature in a shaking water bath with constant stirring. Fallowing incubation, the starch suspension was centrifuged at 1620 *x g* for 10 min. The supernatant was discarded, and the pellet was weighed after draining.

### Solubility and Swelling Capacity (SC)

To determine solubility and swelling capacity, 0.2 g of SLC was suspended in 9.8 mL of distilled water and heated in a water bath at 55℃ and 75 ℃ for 1 h with constant stirring. Subsequently, samples were cooled to room temperature, and centrifuged at 3000 *x g* for 20 min. The supernatants and pellets were dried separately at 105^o^C to constant weight. Solubility and swelling power were calculated by Eq. 7 and Eq. 8 [17]:

7 $$\:Solubility\:\left(\%\right)=\frac{Weight\:of\:dried\:supernetant}{Sample\:of\:weight}\times\:100$$

8 $$\begin{aligned}\:Swelling\:capacity\:\left(\%\right)&=\\\frac{Weight\:of\:wet\:sediment}{Sample\:of\:weight\:times(100\%-solubility\%)}\\times\:100&\end{aligned}$$

### Thermal Properties

Differential Scanning Calorimeter (DSC) (DSC25, TA Instruments, USA) was applied to determination of thermal properties of SLC. For this purpose, the starch sample was mixed with 2 times the amount of water and kept at 4 ℃ for 24 h before analysis. After that, nearly 10 mg sample was weighed into an aluminium DSC pan and pans were hermetically sealed. The changes in thermal properties were recorded at 25–180 °C with 10 °C/min heating rates. An empty pan was used as a reference [18].

### X-Ray Diffraction

X-ray diffraction (XRD) patterns of SLC were analysed by X-ray diffractometer (Bruker D8 Advance, Karlsruhe, Germany) operated at 40 kV and 40 mA. The analyse was performed at a scanning speed of 7°/min and a step size of 0.05° in the 2θ = 5°–40° range [18].

### Statistical Analysis

Starch complexation and analysis were conducted as two replicates. After variance analysis on the data, the Duncan’s Multiple-Range Test (*p* < 0.05) was used to determine the acceptable mean separation. SAS Statistical Software (SAS Institute Inc., Cary, NC, USA) was used for all statistical computations.

## Results and Discussion

### The Changes in Free Fatty Acid, Peroxide and *p*–Anisidine Value of Oils

The changes in free fatty acid content (FFA)(%), peroxide and *p*-anisidine value of raw and hydrolysed sunflower, olive and flaxseed oil are presented in Table 1. The FFA content of raw oils exhibited variability between 0.56 and 2.80% before enzymatic treatment, and it significantly increased to 43.32–49.42% following enzymatic treatment. These results suggested a substantial conversion of triacylglycerol forms of oils into the FFAs with Amano lipase A enzyme utilisation. Additionally, the variety of oil did not exhibit a significant impact on FFA content increase. The effectiveness of lipases in catalysing the conversion of fatty acids depends on both the nature of the oil and the characteristics of the enzyme used. In our study, the maximum conversion level was achievable at approximately 49.42%. The previous studies have been reported various FFA conversion ratio. For example, Di Marco, Ixtaina [13] found a FFA content of 78% by hydrolysis of chia seed oil using *Candida rugosa* originated lipase. Sande, Colen [19] noted the hydrolysation rate by olive and flaxseed oil at the level of 100 and 46% by using *Colletotrichum gloeosporioides* lipase. In another study, sunflower and olive oil could be hydrolysed to yield of 38.32 and 39.74%, respectively, within 24 h by lipase derived from *Aspergillus niger* originated lipase [20].

**Table 1:** Chemical stability of enzymatically hydrolysed oils

Content	Sunflower oil		Olive oil		Flaxseed oil	
	Raw	Hydrolysed	Raw	Hydrolysed	Raw	Hydrolysed
Free fatty acid (%)	0.56 ± 0.00	43.32 ± 0.76	2.80 ± 0.00	49.42 ± 2.66	0.56 ± 0.00	45.92 ± 3.36
Peroxide (mE O_2_/kg)	0.90 ± 0.10	1.00 ± 0.00	2.40 ± 0.00	2.20 ± 0.20	0.30 ± 0.10	0.40 ± 0.00
p-anisidine (mE/kg)	4.31 ± 0.71	6.17 ± 0.07	6.55 ± 0.05	4.95 ± 0.15	nd*	nd*
Fatty acid composition (% area)						
C16:0	6.93±0.1	9.76±0.1	18.77±0.2	21.66±0.4	6.20±0.1	7.28±0.2
C18:0	2.32±0.0	3.35±0.1	3.15±0.1	nd*	3.85±0.1	4.22±0.1
C18:1	30.00±0.4	29.65±0.3	67.07±0.7	66.42±0.5	nd*	nd*
C18:2	60.74±0.5	56.49 0.8	8.77±0.2	5.94±0.1	15.21±0.2	15.31±0.2
C18:3	nd*	0.39±0.1	3.45±0.1	4.30±0.1	74.56±0.6	73.03±0.7

*nd: Not detected. Mean values ± standard deviation (*n* = 2)

Fallowing enzyme treatment, peroxide values of oils remained relatively constant, ranging between 0.90 and 2.40 mE O_2_/kg. The variations in peroxide level were determined due to the oil source. Olive oil exhibited the highest value as 2.40 mE O_2_/kg for raw and 2.20 mE O_2_/kg for hydrolysed oil. These findings are particularly noteworthy since higher peroxide values are mostly associated with oxidation. The results indicate that enzyme treatment can enhance the formation of FFAs without promoting accumulation of oxidative precursors.

The effect of enzymatic hydrolysation had a negligible effect on the *p*-anisidine value, which was generally influenced by the source of oil. A slight increase in the *p*-anisidine value was measured in sunflower (from 4.31 to 6.17 mE/kg) whereas hydrolysis led to a decrease in olive oil (6.55 to 4.95 mE/kg). Additionally, the *p*-anisidine value was below detectable levels both in raw and hydrolysed flaxseed oil.

### The Changes in Fatty Acid Composition of Oils

The fatty acid profile (Table 1) revealed that the major fatty acids presenting in raw and hydrolysed oil samples were palmitic, stearic, oleic, linoleic and linolenic acids. It was concluded that enzyme treatment did not lead to significant differences in the amount of palmitic acid, which constituted 6.21–21.66% of total fatty acid profile in both raw and hydrolysed oils samples. Enzymatic hydrolysis had a significant effect on the stearic acid content of sunflower and flaxseed oil. On the other hand, it was completely lost in olive oil after enzyme treatment, while comprising 3.15% in raw olive oil samples.

Oleic, linoleic and linolenic acids are the primary fatty acids in olive, sunflower and flaxseed oil, respectively. Enzymatic hydrolysis of oils exhibited a negligible effect on the composition of these fatty acids. Similar findings were also declared by Di Marco, Ixtaina [13] who reported hydrolysis was not modified the original fatty acid profile of the chia seed oil. The highest oleic acid content was determined in both raw and hydrolysed olive oil, accounting for 67.07 and 66.42%, respectively. Notably, it was undetected flaxseed oil samples. The greatest amount of linoleic acid was found in raw (60.74%) and hydrolysed (56.49%) sunflower oil. Similarly, the highest level of linolenic acid was detected in raw (74.56%) and hydrolysed flaxseed oil (73.03%), in significant amounts. Additionally, a slight increase in the amount of linolenic acid was observed in sunflower oil following enzymatic hydrolysis.

Based on these findings, it can be concluded that although there was minor variance in the levels of fatty acid, the composition of main fatty acids remained stable following the application of enzyme in sunflower, olive, and flaxseed oil. In this context, these results suggest that enzymatic hydrolysis preserved the fatty acid composition of oils, increased FFAs and did not increase the levels of oxidation indicator compounds.

### In Vitro Starch Hydrolysis

**Fig. 1:**
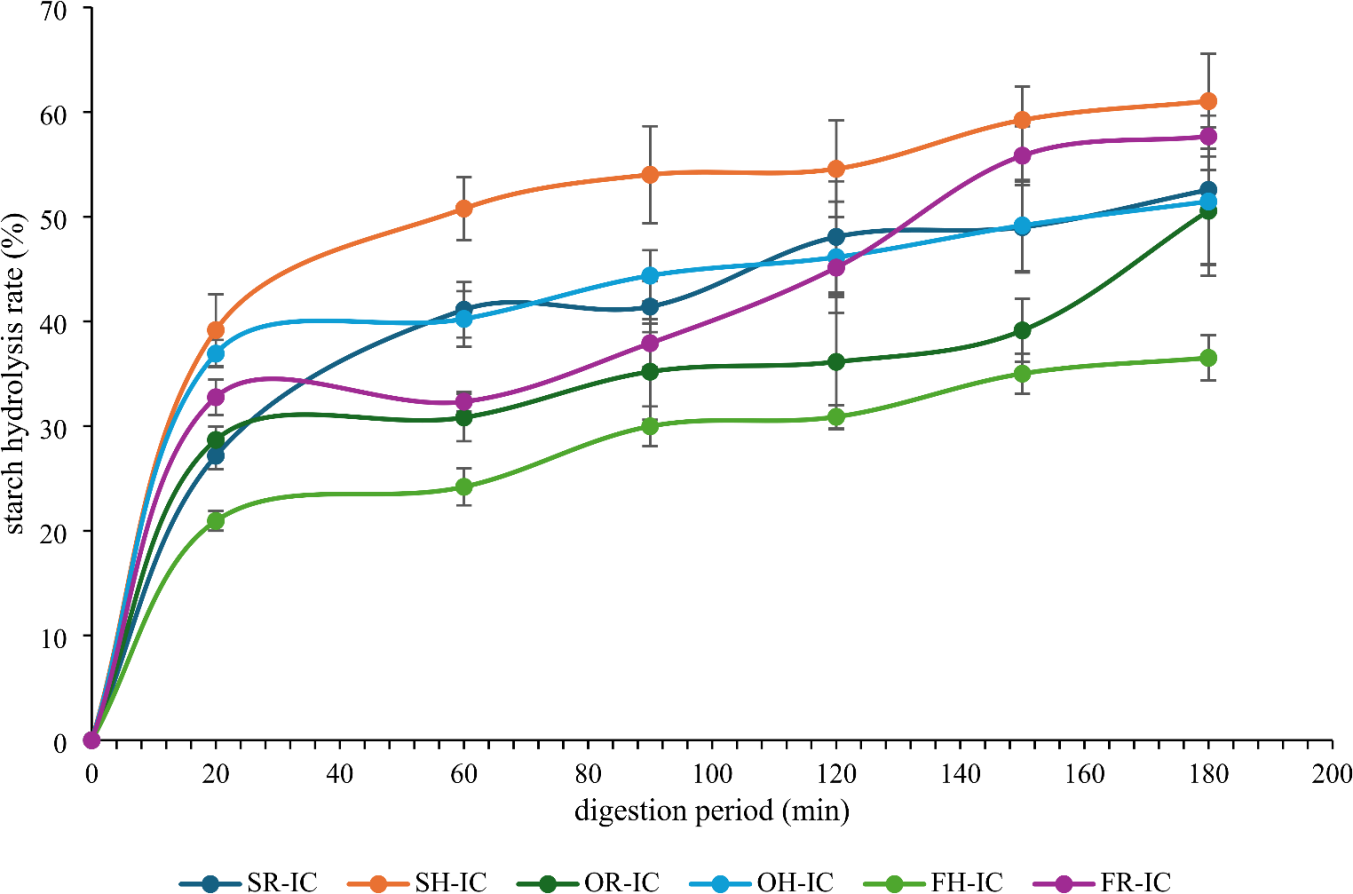
Starch hydrolysis rate. SR-IC: Starch inclusion complex formed with raw sunflower oil, SH-IC: Starch inclusion complex formed with enzymatically hydrolysed sunflower oil, OR-IC: Starch inclusion complex formed with raw olive oil, OH-IC: Starch inclusion complex formed with enzymatically hydrolysed olive oil, FR-IC: Starch inclusion complex formed with raw flaxseed oil, FH-IC: Starch inclusion complex formed with enzymatically hydrolysed flaxseed oil

The hydrolysis rate of SLC synthesised by raw or hydrolysed oils are present in Fig. 1. It was found that SCL with hydrolysed sunflower oil had a comparatively higher degree of hydrolysis than other oils, with a value of 61.03%. This hydrolysis rate was also higher than Hylon VII. Zhai, Zhang [21] also reported that hydrolysation degree and RS content of high amylose starch was higher than SCL produced by stearic acid. The authors attributed this to the higher digestibility of V-type starch compared to B-type starch. Enzymatic hydrolysis of olive oil caused a negligible discrepancy in the starch hydrolysis of SLC, with the hydrolysis level of 50.56 and 51.44%. Most promising results were obtained with flaxseed oil. The use of enzymatically hydrolysed flaxseed oil in the production of complex demonstrated a significant reduction in the starch hydrolysis rate. After 180 min test period, the starch hydrolysis level was 36.52% for the starch complex treated with hydrolysed flaxseed oil. Furthermore, this was the lowest level of starch hydrolysis observed among all samples. From these data it could be concluded that the highly unsaturated fatty acids in flaxseed oil enhance the formation of stronger, more stable SLC. These complexes may limit enzymatic access to the starch molecules, reducing hydrolysis rates and overall digestibility. Unsaturated fatty acids have the potential to modify the structure of starch molecules, enhance the creation of aggregates, and impact the digestibility of starch [4].

In starch hydrolysis, mainly three parts of starch are formed: rapidly digestible starch (RDS), slowly digestible starch (SDS), and resistant starch (RS). According to the Table 2, it was detected that starch complexes produced with hydrolysed oils contained relatively less RDS. Such a diminished RDS content, coupled with elevated SDS levels, represents desirable parameters, as they are generally associated with reduced glycemic response in food products. Additionally, the utilisation of enzymatically hydrolysed oils yielded an insignificant effect on SDS content (*p* > 0.05). The RS content was affected significantly from oil type rather than hydrolysation (*p* < 0.05). The RS content of the samples varied between 45.43 and 69.11%. The highest RS content was obtained with SLC formatted by hydrolysed flaxseed oil. This result was an evident of that higher degree of unsaturation in fatty acids may enhance the interaction with amylose. Similar findings was also reported by Annor, Marcone [22] who investigated the effect of unsaturation level on starch hydrolysis, and the authors found that linolenic acid ensured a higher RS content in all starch sources with a level of 34.7–40.8%. In the another study, Sun, Jin [5] declared that there was a positive correlation between RS content of a complex and the degree of unsaturation of the fatty acids involved complexation structure. In addition, in accordance with the rate of starch hydrolysis, the lowest RS value was determined in the SLC containing sunflower. It is also noteworthy that more than 45% RS was detected in all starch samples irrespective of the enzymatic treatment. As a result of this study, it could be concluded that complexation with both raw and enzymatically hydrolysed oils effectively increased the RS content.

**Table 2:** Rapidly digestible starch (RDS), slowly digestible starch (SDS), resistant starch (RS), and glycemic index (GI) levels of starch inclusion complexes

Starch sample	RDS (%)	SDS (%)	RS (%)	GI
SR-IC	3.28 ± 0.48c	20.91 ± 2.83a	51.93 ± 3.75c	64.23 ± 1.41b
SH-IC	1.68 ± 0.12c	15.40 ± 0.84ba	45.43 ± 3.26c	70.27 ± 2.40a
OR-IC	16.15 ± 1.56a	7.46 ± 0.46b	63.87 ± 4.54ba	60.69 ± 1.07bc
OH-IC	3.08 ± 2.01c	9.21 ± 0.57b	53.87 ± 3.85bc	65.55 ± 1.90ba
FR-IC	13.92 ± 0.45a	12.38 ± 0.80ba	54.86 ± 2.00bc	63.48 ± 0.82b
FH-IC	8.78 ± 0.02b	9.94 ± 0.14b	69.11 ± 0.80a	56.44 ± 0.66c

SR-IC: Starch inclusion complex formed with raw sunflower oil, SH-IC: Starch inclusion complex formed with enzymatically hydrolysed sunflower oil, OR-IC: Starch inclusion complex formed with raw olive oil, OH-IC: Starch inclusion complex formed with enzymatically hydrolysed olive oil, FR-IC: Starch inclusion complex formed with raw flaxseed oil, FH-IC: Starch inclusion complex formed with enzymatically hydrolysed flaxseed oil. In the same column, the superscript letters indicate that they are significantly different by Duncan’s multiple range test (*P* < 0.05)

According to the data presented in Table 2, the eGI values of the complexes varied between 56.44 and 70.27. The SLC formed by hydrolysed flaxseed oil had the lowest eGI. A possible explanation for this result could be the high steric hindrance of linolenic acid, which may render the starch less susceptible to amylolytic enzymes. The complexing capacity may have increased with the degree of unsaturation in the free fatty acids. This finding was supported by previous studies. Annor, Marcone [22] reported that the complexing index increased with the unsaturation of the fatty acid and less starch was hydrolysed in the presence of unsaturated fatty acids. The lowest eGI value was found in cooked maize starches with linolenic acid [22].

### Water Holding Capacity (WHC), Solubility and Swelling Capacity of Starch Complex

The changes in the physical properties of starch by complexation with raw and hydrolysed oils are depicted in Table 3. Based on the WHC findings, SLCs containing hydrolysed fatty acids had higher WHC, except for those involving flaxseed oil. There was no statistically significant difference in the WHC between complexes formed by both raw and hydrolysed flaxseed oil, with respective values of 280.95 and 284.20%. This result might be attributed to the high content of unsaturated ester bonds and bending zones in flaxseed oil, which may increase the interaction between water and complexes. Research suggests that the water-holding capacity of starch-lipid complexes formed by unsaturated fatty acids can be influenced by several factors. Wang, Wu [23] further demonstrated that the water content of the starch can also affect the formation of these complexes, with higher water content leading to a higher complex index. Moreover, the WHC of Hylon IIV was determined to be 95% after testing. This outcome demonstrates that starch complexation with both raw and hydrolysed oils enhanced water retention within the polymeric structure. Furthermore, high WHC of FH-IC may be associated with a denser microstructure and a lower degree of starch gelatinisation, resulting in reduced in vitro starch digestibility. This effect was also observed in the study by Zhang, Zeng [24] who determined that the incorporation of hydrocolloid (konjac glucomannan) into starch-based systems significantly reduced the starch hydrolysis rate. This effect was attributed to high WHC, which interferes with starch gelatinisation and enzyme accessibility, thereby reducing digestibility.

**Table 3:** Physical properties of starch inclusion complexes

Starch sample	Water holding capacity (%)	Solubility (%)		Swelling capacity (%)	
		55 °C	75 °C	55 °C	75 °C
SR-IC	212.69 ± 6.37c	0.77 ± 0.07c	2.58 ± 0.12ba	5.16 ± 0.06bc	5.85 ± 0.06d
SH-IC	242.44 ± 8.01cb	1.21 ± 0.01a	3.44 ± 0.31a	5.11 ± 0.14c	5.88 ± 0.01d
OR-IC	247.64 ± 16.71cb	1.23 ± 0.04a	1.80 ± 0.24bc	5.78 ± 0.25a	6.32 ± 0.18c
OH-IC	237.98 ± 10.06b	0.94 ± 0.12bc	1.61 ± 0.38bc	5.63 ± 0.06ba	6.86 ± 0.01b
FR-IC	284.20 ± 1.69a	1.15 ± 0.08ba	2.10 ± 0.28bc	5.77 ± 0.22a	7.28 ± 0.01a
FH-IC	280.95 ± 5.53a	0.81 ± 0.08c	1.36 ± 0.49c	5.74 ± 0.05a	7.49 ± 0.10a
Hylon VII	95.12 ± 0.03d	0.00 ± 0.00d	2.08 ± 0.40bc	2.23 ± 0.09d	3.40 ± 0.16e

SR-IC: Starch inclusion complex formed with raw sunflower oil, SH-IC: Starch inclusion complex formed with enzymatically hydrolysed sunflower oil, OR-IC: Starch inclusion complex formed with raw olive oil, OH-IC: Starch inclusion complex formed with enzymatically hydrolysed olive oil, FR-IC: Starch inclusion complex formed with raw flaxseed oil, FH-IC: Starch inclusion complex formed with enzymatically hydrolysed flaxseed oil. In the same column, the superscript letters indicate that they are significantly different by Duncan’s multiple range test (*P* < 0.05)

The solubility (%) of the starch complex was assessed at two different temperatures. The solubility of the complex ranged from 0.77 to 1.23% at 55 °C and from 1.36 to 3.44% at 75 °C. Higher testing temperatures were found to enhance the solubility of the SLC. The utilisation of enzymatically hydrolysed forms of oils resulted in decreased solubility except sunflower oil. Specifically, the high solubility values were determined with SH-IC to be 1.21% and 3.44% in tested temperatures. On the other hand, the lowest solubility values were detected with FH-IC despite their high WHC. This finding may be linked to the high RS content of FH-IC which could decrease solubility due to increased crystallinity of structure. Additionally, solubility of Hylon IIV was totally non-soluble at 55 °C and its solubility degree was 2.08% at 75 °C. The fact that the starch granules were not structurally disrupted by the pre-gelatinisation of Hylon IIV may explain this result.

As expected, contrasting outcomes were obtained regarding swelling capacity compared to solubility. Starch complex with the highest solubility, SH-IC, exhibited the lowest swelling capacity at the tested temperatures. Moreover, the raise of temperature lead to increase swelling capacity of starch complex and, the highest swelling capacity recorded for the complex containing flaxseed oil at 7.28% and 7.49% at 75 °C. This finding was also consistent with the findings of WHC. Thus, it can be inferred that there exists a positive correlation between WHC and swelling capacity whereas solubility demonstrates a negative correlation. Furthermore, the swelling capacity of Hylon IIV was determined to be 2.23% at 55 °C and 3.40% at 75 °C. These results suggests that the complexation of native starch with both raw and hydrolysed oils lead to increase swelling power.

### Thermal Properties

DSC characteristics of starch complexes are presented in Table 4. The utilisation of raw and enzymatically hydrolysed oils had an insignificant effect on onset temperatures (*p* > 0.05) with start of melting at 98 °C for all SLCs. Additionally, the highest melting temperature (T_peak_) were measured in SLC utilised by hydrolysed olive and flaxseed oil. In general, raw oil usage in formation of SLC lead to decrease T_peak_. It is noteworthy that T_peak_ of the complexes were very close to each other, despite the presence of statistical differences. This outcome could be attributed to the fact that the major fatty acid involved in the formation of complexes possesses same chain lengths. This result aligns with previous studies, indicating a direct corelation between the chain length of fatty acids and higher melting temperatures. Similar results were also obtained by Sun, Jin [5] who identified endothermic peaks within the range of 87.30 to 127.65 °C in the thermogram of SLCs. They correlated the variation in T_peak_ with the length of the fatty acid chain and degree of unsaturation. Furthermore, these high T_peak_ of SLC formed by unsaturated fatty acid was addressed as a good vehicle of poly unsaturated fatty acid in thermally-treated foods [13].

**Table 4:** Thermal properties of starch inclusion complexes

Starch sample	T_o_	T_e_	T_*p*_	T_e_-T_o_	PHI	ΔH j/g
SR-IC	98.16 ± 0.16a	144.29 ± 0.28b	126.42 ± 0.20a	46.13 ± 0.62	28.11 ± 0.10	1269.74 ± 15.28a
SH-IC	98.21 ± 0.21a	148.34 ± 0.34a	123.25 ± 0.05b	50.13 ± 0.78	24.92 ± 0.23	1174.80 ± 4.80b
OR-IC	98.02 ± 0.03a	145.12 ± 0.02b	124.09 ± 0.02b	47.10 ± 0.01	27.90 ± 0.51	1316.89 ± 16.89a
OH-IC	98.02 ± 0.02a	142.40 ± 0.30c	127.25 ± 0.13a	44.38 ± 0.01	25.16 ± 0.53	1116.62 ± 16.62b
FR-IC	97.89 ± 0.11a	143.05 ± 0.15c	123.17 ± 0.10b	45.16 ± 0.06	29.04 ± 0.32	1311.52 ± 11.51a
FH-IC	98.08 ± 0.08a	142.19 ± 0.11c	127.42 ± 0.28a	44.11 ± 0.27	30.24 ± 1.15	1334.02 ± 30.0a

SR-IC: Starch inclusion complex formed with raw sunflower oil, SH-IC: Starch inclusion complex formed with enzymatically hydrolysed sunflower oil, OR-IC: Starch inclusion complex formed with raw olive oil, OH-IC: Starch inclusion complex formed with enzymatically hydrolysed olive oil, FR-IC: Starch inclusion complex formed with raw flaxseed oil, FH-IC: Starch inclusion complex formed with enzymatically hydrolysed flaxseed oil. In the same column, the superscript letters indicate that they are significantly different by Duncan’s multiple range test (*P* < 0.05)

Moreover, there are two forms of SLC: semi-crystalline type II complexes (with a melting temperature of approximately 115–130 °C) and less-ordered type I complexes (with a melting temperature of approximately 90–105 °C) [5]. Additionally, type II is mostly associated with higher resistance to amylase enzyme [5, 6]. According to findings, starch complex both produced by raw and hydrolysed oils represented type II complex structure, and high RS content could be associated with this structure.

The values of T_e_–T_o_ reflect the degree of difficulty in gelatinizing the starch granules, which a higher value indicates a more challenging gelatinisation process. In comparison to the control, the utilisation of hydrolysed olive and flaxseed oil yielded similar gelatinisation characteristics. However, with maximum T_e_–T_o_ values, the utilisation of hydrolysed sunflower oil resulted difficulty in starch gelatinization [25]. Another, parameter is the peak high index (PHI) which is used to measure the uniformity in gelatinization [25]. The PHI was similar in the control and SLC produced by hydrolysed flaxseed oil, indicating a higher level of gelatinisation uniformity in the samples.

A more significant result was obtained in terms of the enthalpy of gelatinisation (ΔH). The utilisation of hydrolysed sunflower and olive oil led to a decrease in the ΔH of SLCs. However, this trend could not be observed in the complexes formed by flaxseed, and ΔH was statistically similar in both complexes produced by hydrolysed and non-hydrolysed oils. Additionally, the highest ΔH was measured in FH-IC at 1334.02 J/g. This could be due to the high RS content of this sample.

### XRD Pattern of SLC

**Fig. 2:**
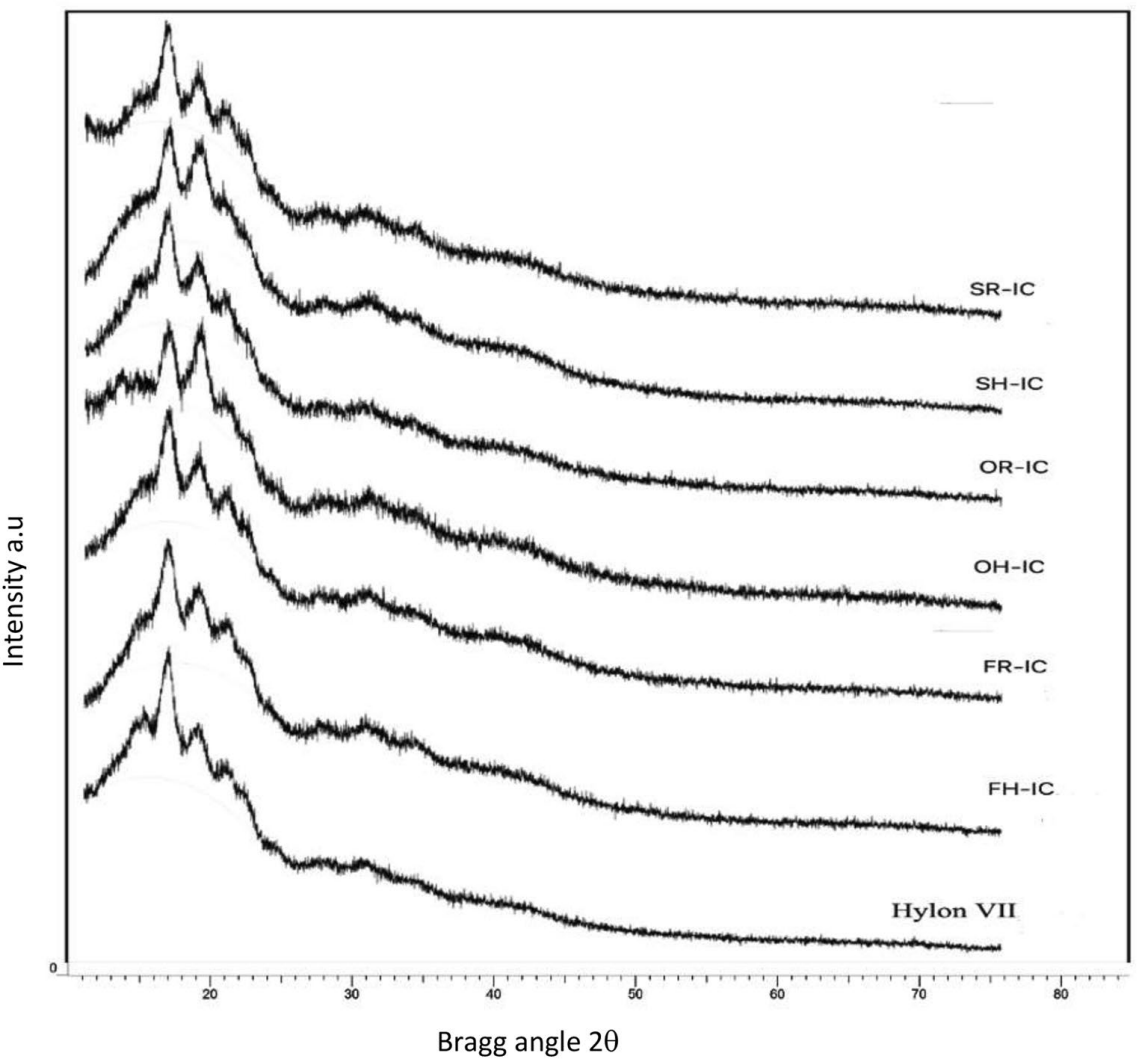
XRD Intensity of starch inclusion complex

XRD patterns of starches vary depending on their sources. A-type starches generally exhibit a pattern with a singlet peak at 2θ = 15°, double peaks at 17° and 18°, and another peak at 23°, while B-type starches consist of peaks at 14°, 15° 22° and 24°. C-type pattern is a mixture of A and B-type patterns, and it is seen in generally in high amylose maize starch [25]. Figure 2 also showed that Hylon IIV contains peaks at 15°, 17°, 20° 22° and 24°. Figure 1 supplementary showed that the XRD patterns of SLC involved by hydrolysed fatty acids had peaks at 2θ = 13°, 17° and 20° which are the typical characteristics of V-type structure [25]. These peaks were more obvious in the samples of OH-IC. On the other hand, V-type formation could not be detected in SLCs produced by raw oils (Fig. 1 supplementary). In the study of Li, Luo [11], both the triglyceride and free fatty acid forms of lauric, myristic, palmitic, and stearic acid were tested for the formation of inclusion complexes. It was determined that V-type formation could only occur in samples containing free fatty acids.

## Conclusion

In this study, the capability of free and triglyceride forms of fatty acids to form inclusion complexes with high amylose maize starch was investigated. The starch hydrolysis rate was significantly affected by the type of oil rather than the form of fatty acids. A significant reduction in the hydrolysis rate was noted by the complexation of starch with hydrolysed flaxseed oil. A high content of RS (≤ 45%) was obtained with all types of SLC. According to the crystallinity model, V-formation was detected only in SCL formed from hydrolysed oil. From this result it can be concluded that the SLCs that were synthesised only from hydrolysed oils can be classified as RS5. Moreover, increases in water holding capacity (WHC) and swelling capacity were observed, particularly in complexes involving hydrolysed oils, suggesting improved moisture retention and structural integrity. Overall, the results highlight the potential of enzymatic hydrolysis of oils to modulate the physicochemical properties of starch complexes, offering opportunities for the development of functional food ingredients with tailored nutritional and functional characteristics. Further research is warranted to explore the applicability of these complexes in various food formulations and their potential health benefits in human consumption.

## Electronic Supplementary Material

Below is the link to the electronic supplementary material.

Supplementary Material 1

## Data Availability

No datasets were generated or analysed during the current study.
