# Supplementary material for: The Formation of Starch-Lipid Inclusion Complex by Enzymatic Hydrolysed Oils
Source: Plant Foods Hum Nutr. 2025 Mar 4;80(1):84. doi: 10.1007/s11130-025-01326-7 (PMC11880067; doi:10.1007/s11130-025-01326-7)
Supplement: Supplementary file 1 — Supplementary Material 1 [file 11130_2025_1326_MOESM1_ESM.docx]

Figure 1 supplementary. Detailed XRD patterns of starch complex and Hylon IIV at 2θ=13°
